# The Highly Conserved Cys95 Residue of Fructose‐1,6‐Bisphosphatase 1 Mediates the pH‐Driven Structure and Activity of the Enzyme and Photosynthesis

**DOI:** 10.1111/pce.15667

**Published:** 2025-06-08

**Authors:** Samuel Gámez‐Arcas, Edurne Baroja‐Fernández, Francisco José Muñoz, Antonio J. Serrato, Mónica Balsera, Ángela María Sánchez‐López, Abdellatif Bahaji, Jesús Leal‐López, Rafael Jorge León Morcillo, Javier Pozueta‐Romero

**Affiliations:** ^1^ Instituto de Agrobiotecnología (IdAB), CSIC‐Gobierno de Navarra Nafarroa Spain; ^2^ Instituto de Bioquímica Vegetal y Fotosíntesis, Consejo Superior de Investigaciones Científicas (CSIC)‐Universidad de Sevilla Sevilla Spain; ^3^ Departamento de Bioquímica, Biología Molecular y Celular de Plantas, Estación Experimental del Zaidín Consejo Superior de Investigaciones Científicas Granada Spain; ^4^ Department of Abiotic Stress Instituto de Recursos Naturales y Agrobiología de Salamanca (IRNASA‐CSIC) Salamanca Spain; ^5^ Institute for Mediterranean and Subtropical Horticulture “La Mayora” (IHSM) CSIC‐UMA, Campus de Teatinos, Avda Málaga Spain

**Keywords:** 6‐bisphosphatase, AlphaFold, Calvin–Benson cycle, fructose‐1, pH and redox regulation, photosynthesis, protein structure

## Abstract

In Arabidopsis, exposure to microbial volatile compounds promotes thiol reduction of the Cys95 residue of the photosynthetic enzyme fructose‐1,6‐bisphosphatase (cFBP1). Although highly conserved in plants, the Cys95 function still remains unknown. We characterised recombinant wild‐type (WT) cFBP1 and a variant (C95S) in which the Cys95 residue was replaced by serine. Furthermore, we characterised cFBP1‐lacking *cfbp1* transgenic plants expressing WT or C95S cFBP1. Cys95 replacement by serine reduced cFBP1 activity and its Mg^2+^ binding affinity and cooperativity. Although it is widely assumed that active cFBP1 is strictly homotetrameric, WT and C95S cFBP1 were present as inactive tetramers at pH 7.0 and active dimers at pH 8.3. At pH 7.8, WT and C95S cFBP1 were predominantly present as dimers and tetramers, respectively. WT cFBP1 expression totally reverted to WT the reduced photosynthetic activity of *cfbp1* plants grown in the absence or presence of microbial volatiles, but that of C95S cFBP1 only partially did it. Artificial intelligence‐based AlphaFold protein structure analyses predicted that the replacement of Cys95 by serine promotes cFBP1 conformational changes. We conclude that (i) active cFBP1 is strictly dimeric at pH values occurring in illuminated chloroplasts and (ii) Cys95 is an important determinant of the stromal pH‐driven structure and activity of cFBP1 and photosynthesis.

## Summary statement

The highly conserved Cys95 of the Calvin–Benson cycle enzyme cFBP1 plays an important role in regulating pH‐driven oligomeric status, activity and conformational state of the Mg^2+^ and substrate binding domains of the enzyme and photosynthesis in Arabidopsis.

## 1 Introduction

cFBP1 is a metal (Mg^2+^)‐requiring enzyme of the Calvin–Benson cycle (CBC) that catalyses the hydrolytic breakdown of fructose‐1,6‐bisphosphate (FBP) to fructose‐6‐phosphate and Pi. Like in mammalian gluconeogenic fructose‐1,6‐bisphosphatases (FBPase), crystallographic studies showed that active cFBP1 is a strictly homotetrameric enzyme that exists in distinct and allosterically regulated quaternary ‘relaxed’ (R, active) and ‘tense’ (T, inactive) states (Ke et al. 1990; Xue et al. 1994; Villeret et al. 1995; Chiadmi 1999; Gütle et al. 2016). It behaves as a hysteretic enzyme that exhibits slow pH‐dependent conformational changes (Zimmermann et al. 1976; Pradel et al. 1981; Gontero et al. 1984; Ballicora and Wolosiuk 1990). cFBP1 is activated by light through mechanisms involving chloroplast alkalinization, enhanced Mg^2+^ concentration and reduction of disulphide groups of cysteine (Cys) residues via the ferredoxin (Fdx)/thioredoxin *f* (Trx *f*) and NTRC systems (Zimmermann et al. 1976; Hertig and Wolosiuk 1983; Cejudo et al. 2021; Yoshida and Hisabori 2023). In addition, cFBP1 can be inactivated by *S‐*nitrosylation of Cys residues subjected to Fdx/Trx *f* redox modifications (Serrato et al. 2018). Plant cFBP1 monomers contain seven highly conserved Cys residues, which in Arabidopsis are located at positions 52, 95, 156, 174, 179, 191 and 207 of the mature protein (Figure S1) (Villeret et al. 1995; Rodríguez‐Suárez et al. 1997). Four of these residues (e.g. Cys95, Cys156, Cys174 and Cys179) are not present in the cytosolic isoform of plant FBPase (CyFBP) and in mammalian gluconeogenic FBPases (Figure S1), which would indicate that they play important roles in the regulation of cFBP1 activity. Crystallographic and activity studies using site‐directed mutated pea and rapeseed cFBP1 showed that Cys156, Cys174 and Cys179, but not Cys95, mediate the redox regulation of cFBP1 by the Fdx/Trx *f* system (Villeret et al. 1995; Jacquot et al. 1997; Rodríguez‐Suárez et al. 1997; Chiadmi 1999; Serrato et al. 2018). Furthermore, data obtained from studies using site‐directed mutated variants of rapeseed (*Brassica napus*) cFBP1 in which the cysteine residues were replaced by serine (Ser) provided evidence that (i) none of the highly conserved Cys residues are important determinants of the catalytic activity of the enzyme and (ii) Cys52 and Cys191 are important for cFBP1 stability (Rodríguez‐Suárez et al. 1997). Given that Cys95 is not an important determinant of cFBP1 structure, stability and activity (Villeret et al. 1995; Rodríguez‐Suárez et al. 1997), its function and the reasons for its absolute conservation throughout plant evolution still remain unknown (Rodríguez‐Suárez et al. 1997).

Microorganisms release volatile compounds (VCs) of less than 300 Da that promote plant growth, developmental changes and photosynthesis as well as drastic changes in the hormonome, transcriptome, proteome and metabolome (García‐Gómez et al. 2020; Gámez‐Arcas et al. 2022). Some of these compounds are very reactive with proteins and/or act as signalling molecules that promote photosynthesis, growth and developmental changes when exogenously applied in a discrete form and/or in low concentrations (García‐Gómez et al. 2019; Gámez‐Arcas et al. 2022; Morcillo et al. 2024). Redox‐proteomic analyses revealed that microbial VCs induced global reversible thiol redox proteome changes (Ameztoy et al. 2019). These changes included the thiol reduction of highly conserved Cys residues in proteins involved in photochemical reactions and CBC enzymes, including the Cys95 residue of one of the two isoforms of plastid‐localised fructose‐1,6‐bisphosphatase (cFBP1) (Ameztoy et al. 2019). It thus appears that the response of plants to microbial VC is due, at least partly, to redox switching mechanisms that involve rapid thiol redox activation of the photosynthetic machinery.

Although Cys is the second least abundant amino acid in proteins, Cys residues are highly conserved and play important roles in the oligomerization and stabilisation of protein structures, metal binding, redox sensitivity, regulating catalysis, etc. (Martz et al. 2002; Marino and Gladyshev 2010; Bak et al. 2019; Cannon and Horn 2023; Huang et al. 2024). The fact that microbial VC exposure enhances photosynthesis and reduces the thiol of Cys95 of cFBP1 could indicate that this residue plays important roles in the structure and activity of cFBP1, which were overlooked in previous studies. To test this hypothesis, in this study we produced in *Escherichia coli* mature wild‐type (WT) cFBP1 and a mutated form of cFBP1 (C95S) in which the Cys95 residue has been replaced by Ser, and compared their structural and functional properties. We also produced and characterised the photosynthetic activity of cFBP1‐lacking *cfbp1* plants expressing the WT and the C95S forms of cFBP1 under the control of the *cFBP1* promoter (*promcFBP1*). The results presented here provide strong evidence that the highly conserved Cys95 residue of cFBP1 is an important determinant of stromal pH‐dependent cFBP1 conformational status and activity and photosynthesis in Arabidopsis.

## 2 Materials and Methods

### 2.1 Transgenic Plant Production

The work was carried out using *Arabidopsis thaliana* L. (Heynh) WT plants (ecotype Columbia Col‐0), the *cfbp1* knockout mutant, line GK‐472G06‐019879 (Rojas‐González et al. 2015) and *cfbp1* plants transformed with either *promcFBP1:cFBP1*_*WT*_ or *promcFBP1:cFBP1*_*C95S*_, which express the WT and C95S cFBP1 under the control of the 1094 bp *promcFBP1* immediately upstream of the translation start codon of *cFBP1* (Figure S2). To construct *promcFBP1:cFBP1*_*WT*_ and *promcFBP1:cFBP1*_*C95S*_, a complete cDNA corresponding to the WT *cFBP1* gene (At3g54050) was obtained from the RIKEN Arabidopsis cDNA collection (pda00367) (Seki et al. 1998, 2002), amplified by PCR using the specific ‘attB1 *cFBP1*’ and ‘attB2 *cFBP1*’ primers (Table S1) and cloned into the pDONR/Zeo plasmid using BP clonase (Invitrogen). The *cFBP1*_*C95S*_ cDNA was generated by site‐directed mutagenesis using the plasmid pDONR‐cFBP1_WT_, the oligonucleotides 5′‐ctaatgaggtgttttccaactctttgagatcaagtggaagaac‐3′ and 5′‐ gttcttccacttgatctcaaagagttggaaaacacctcattag‐3′, and the QuickChange Site‐directed Mutagenesis Kit (Stratagene) to replace the codon initially encoding Cys95 by a codon encoding Ser95. The pDONR‐cFBP1_WT_ and pDONR‐cFBP1_C95S_ entry clones were then subjected to LR recombination with pDONR4‐1R cFBP1 promoter using the R4pGWB501 destination vector, respectively. All plasmid constructs were electroporated and propagated in *E. coli* TOP 10. The final destination vectors were confirmed by sequencing, transferred to *Agrobacterium tumefaciens* EHA105 cells by electroporation and utilised to transform *cfpb1* Arabidopsis plants using the floral dip method described by Clough and Bent (1998). Plants were selected on hygromycin‐containing medium, and six independent homozygous lines of cfbp1 plants transformed with either promcFBP1:cFBP1WT or promcFBP1:cFBP1C95S were obtained and confirmed by PCR.

### 2.2 Plants, Growth Conditions and Sampling

Plants were grown on soil or in Petri dishes (92 × 16 mm, Sarstedt, Ref. 82.1472.001) containing half‐strength agar solidified Murashige and Skoog (MS) (Phytotechlab M519) medium in growth chambers with a 16 h light (22°C, 90 µmol photons sec^−1^ m^−2^)/8 h dark (18°C) cycles. Fully expanded source leaves of plants grown on soil were harvested, immediately frozen, and ground in liquid nitrogen. To investigate the effects of small fungal VCs on plants, we used the ‘plasticized PVC wrap and charcoal filter‐based box‐in‐box’ cocultivation system (Gámez‐Arcas et al. 2022). *A. alternata* was grown in Petri dishes containing agar solidified MS medium supplemented with 90 mM sucrose.

### 2.3 Production of Recombinant WT and C95S cFBP1 Mature Forms in *E. coli* and Purification

*E. coli* TOP 10 was used for gene cloning, and BL21(DE3) was employed for expression of recombinant predicted mature forms of WT (cf. Figure S1) and C95S cFBP1. pDEST17‐cFBP1_WT_ and pDEST17‐cFBP1_C95S_ plasmids used to express mature forms of WT and C95S cFBP1 were produced using the Gateway technology as shown in Figure S2 and confirmed by sequencing. Briefly, cDNAs from *cFBP1*_*WT*_ and *cFBP1*_*C95S*_ were obtained by PCR using pDONR‐cFBP1_WT_ and pDONR‐cFBP1_C95S_ as templates, respectively, and the specific primers ‘attB1 cFBP1*’ and ‘attB2 cFBP1’ (Table S1), and cloned into the pDONR/Zeo plasmid using BP clonase (Invitrogen). To produce pDEST17‐cFBP1_WT_ and pDEST17‐cFBP1_C95S_, pDONR cFBP1_WT_ and pDONR‐ cFBP1_C95S_ entry clones were subjected to LR recombination using the pDEST17 destination vector, respectively. All final destination vectors were confirmed by sequencing. BL21(DE3) cells transformed with pDEST17‐cFBP1_WT_ and pDEST17‐cFBP1_C95S_ were grown at 37°C in 1 L of LB medium supplemented with 50 µg mL^−1^ carbenicillin to an absorbance at 600 nm of 0.6, and then 0.4 mM isopropyl‐β‐d‐thiogalactopyranoside was added to the culture medium. After 3 h, the bacterial culture was centrifuged at 6000 × *g* for 10 min. The pelleted bacteria were resuspended in 6 mL of HiTrap TALON crude binding buffer (GE Healthcare Life Sciences, Marlborough, MA, USA), sonicated, and centrifuged at 10,000 × *g* for 10 min. The supernatant thus obtained was subjected to Co^2+^ affinity chromatography according to the manufacturer's instructions (GE Healthcare Life Sciences). The eluted hexahistidine‐tagged recombinant proteins were then stored in small aliquots at −80°C at a concentration of ca. 1.0 mg/mL.

### 2.4 Determination of FBPase Activity

We used the spectrophotometric assay method described in Sahrawy et al. (2022). The assay was conducted using microtiter plates in a final volume of 200 µL. Unless otherwise indicated, the reaction mixture contained 100 mM Tris–HCl pH 8.5, 10 mM MgCl_2_ (or a variable concentration of MgCl_2_ for *K*_m_ determination), 100 mM DTT, 0.3 mM NADP, 12 mM FBP (or a variable concentration for *K*_m_ determination), 0.7 U of glucose‐6‐P dehydrongease (ROCHE, Basel, Switzerland) and 0.3 U of phosphoglucose isomerase (ROCHE). The increase of absorbance at 340 nm (NADPH formation) vs. time was read with a microplate reader Tecan Sunrise (Tecan Trading AG, Männedorf, Switzerland). Incubations and activity assays were performed for 5 min at room temperature (22°C–24°C). A plot of velocity vs. substrate concentration was used in the *K*_m_ determination. Curve fitting and determination of enzyme parameters were performed using R software (RStudio Team (2020). RStudio: Integrated Development for R. RStudio, PBC, Boston, MA. Available online: http://www.rstudio.com/). One unit (U) of enzyme activity was defined as the amount of enzyme required to hydrolyse 1 µmol of FBP per minute.

### 2.5 Western Blot Analyses

For immunoblot analyses, protein samples were separated on 10% SDS‐PAGE, transferred to PVDF, and immunodecorated by using the antisera raised against cFBP1 from pea (Rojas‐González et al. 2015) and a goat anti‐rabbit IgG horseradish peroxidase conjugate as secondary antibody (Sigma).

### 2.6 Analytical Procedures

Protein content was determined using the Bradford method using Bio‐Rad prepared reagent (Bio‐Rad Laboratories, Hercules, CA, USA). The native molecular masses of recombinant WT and C95S cFBP1 were determined by gel filtration on a Superdex 200 HR 10/30 column (Pharmacia LKB) with 50 mM Tris (pH 7.0, 7.8 or 8.3)/0.15 M NaCl using an AKTA FPLC, a Frac‐950 Fraction Collector (Amersham Pharmacia Biotech) and a Bio‐Rad Kit of protein standards (bovine thyroglobulin: 669 kDa; bovine γ‐globulin: 158 kDa; chicken ovoalbumin: 44 kDa; horse myoglobin: 17 kDa) (gel filtration standard 1511901). The elution was carried out at a flow rate of 0.3 mL/min, and 0.15 mL fractions were collected.

### 2.7 Determination of Gas Exchange Rates

Gas exchange rates were determined as described by Sánchez‐López et al. (2016) using a LI‐COR 6400 gas exchange portable photosynthesis system (LI‐COR, Lincoln, NE, USA).

### 2.8 Modelling of cFBP1 Structure

The crystal structures of the pea and spinach cFBP1 tetramers are available in the Protein Data Bank (https://www.rcsb.org/structure/1D9Q#entity-1; https://www.rcsb.org/structure/1SPI#entity-1). The predicted structures of WT and C95S forms of Arabidopsis cFBP1 were obtained through the AlphaFold3 Protein Structure Database (https://www.alphafold.ebi.ac.uk) (Jumper et al. 2021; Varadi et al. 2022).

### 2.9 Statistical Analysis

Unless otherwise indicated, the presented data on plant growth and photosynthetic activities are means (±SE) obtained from three to four independent experiments, with three replicates for each experiment. The significance of differences was statistically evaluated with Student's *t*‐test using SPSS software. Differences were considered significant at a probability level of *p* < 0.05. For kinetic constants of recombinant mature WT and C95S cFBP, the non‐parametric Mann–Whitney *U*‐test was applied to evaluate statistical differences between enzymatic parameters.

## 3 Results

### 3.1 Replacement of Cys95 by Serine Reduces the Activity and Alters the pH‐Dependent Structure of cFBP1

**Figure 1:**
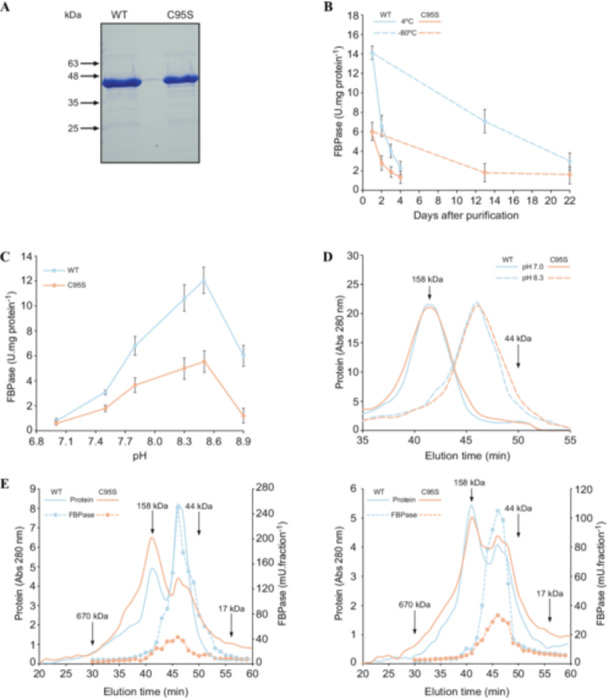
Characterisation of recombinant WT and C95S cFBP1. (A) SDS‐PAGE and Coomassie blue staining of purified WT and C95S cFBP1. (B) Time‐course analysis of FBPase activities of WT and C95S cFBP1 preparations stored for the indicated times at 4°C and −80°C. (C) pH‐dependent FBPase activities of WT and C95S cFBP1. (D) Elution profiles of recombinant WT and C95S cFBP1 subjected to gel filtration on a Superdex 200 column equilibrated with 50 mM Tris, pH 7.0/0.15 M NaCl or 50 mM Tris, pH 8.3/0.15 M NaCl. (E) Elution and activity profiles of recombinant WT and C95S cFBP1 fresh preparations (left panel) and the same preparations stored at 4°C for 1 day (right panel) subjected to gel filtration on a Superdex 200 column equilibrated with 50 mM Tris pH 7.8/0.15 M NaCl.

In routine protein mutation studies, Cys is preferentially mutated to Ser or alanine (Ala) (Rodríguez‐Suárez et al. 1997; Martz et al. 2002; Decker et al. 2023). These replacements are thought to be the most appropriate in suppressing sulphur chemistry while not influencing protein structure. To investigate the possible dependence of cFBP1 activity, stability and structure on Cys95, we assayed activities at saturating substrate (FBP and Mg^2+^) concentrations and the molecular masses of recombinant mature WT and C95S cFBP1 at different pH values and over time in preparations stored at 4°C and −80°C. As shown in Figure 1A, the recombinant cFBP1 preparations were homogeneous when analysed by SDS‐PAGE and Coomassie blue staining, exhibiting single bands whose molecular masses of ca. 40 kDa corresponded to that of the mature cFBP1 of Arabidopsis. Notably, freshly prepared WT cFBP1 was ca. 2.5‐fold more active than C95S cFBP1 (Figure 1B). In keeping with Zimmermann et al. (1976) and Jacquot et al. (1995), WT cFBP1 activity declined within a few days of storage at 4°C and less rapidly at −80°C, reaching values comparable to those of C95S cFBP1 (Figure 1B). Both WT and C95S cFBP1 were devoid of activity at pH 7, but their activities increased with pH values ranging from 7.5 to 8.5 and then drastically decayed with pH values higher than 8.5 (Figure 1C), which is consistent with Ballicora and Wolosiuk (1990) and Serrato et al. (2009). In gel filtration experiments at pH 7.0, both WT and C95S cFBP1 eluted as ca. 160 kDa, indicating a tetrameric state (Figure 1D). In contrast, the two cFBP1 variants eluted as ca. 80 kDa dimers at pH 8.3 (Figure 1D). At pH 7.8, freshly prepared WT and C95S cFBP1 eluted predominantly as active dimers and inactive tetramers, respectively (Figure 1E, left panel). After 1 day of storage at 4°C, both WT and C95S cFBP1 eluted predominantly as inactive tetramers (Figure 1E, right panel). The specific activity of the WT cFBP1 dimeric form was ca. threefold higher than that of C95S cFBP1 (Figure 1E).

### 3.2 Replacement of Cys95 by Serine Reduces the Mg^2+^ Cooperativity of cFBP1

**Figure 2:**
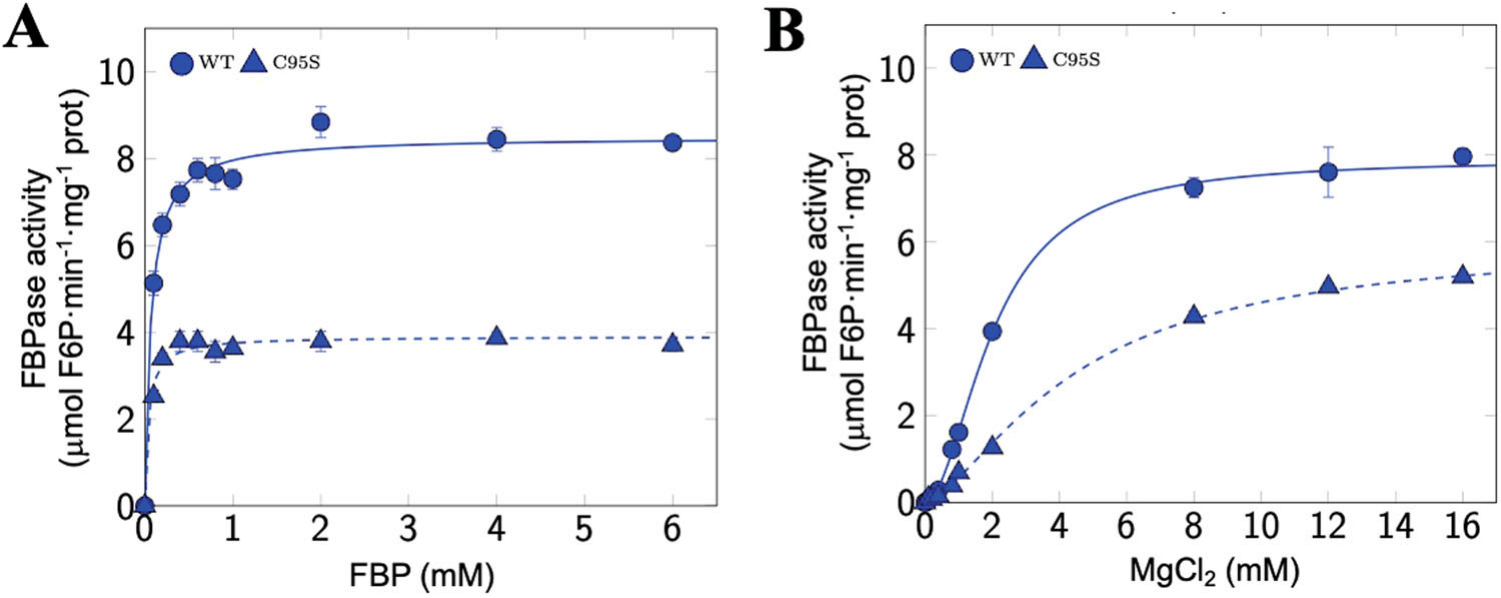
Replacement of Cys95 by serine reduces the activity and Mg2+ cooperativity of cFBP1. The graphs represent the fructose‐1,6‐bisphosphatase (FBPase) activity curves with respect to (A) fructose‐1,6‐bisphosphate (FBP) and (B) Mg2+ concentrations. The mean of three enzymatic assays is represented. [Color figure can be viewed at wileyonlinelibrary.com]

Whether Cys95 plays an important role in cFBP1 activity was further investigated by characterising the kinetic properties of freshly prepared recombinant mature WT and C95S cFBP1 at pH 8.5 using different concentrations of FBP and Mg^2+^ (Figure 2). *K*_cat_ of the C95S cFBP1 (10.7 ± 0.2 s^−1^) was substantially lower than that of WT (23.5 ± 0.3 s^−1^) (Table 1). Under these conditions, WT and C95S cFBP1 exhibited Michaelis–Menten kinetics with respect to FBP (Figure 2), with *K*_m_ values of 68.0 ± 3.0 and 41.0 ± 4.0 µM, respectively (Table 1). Mg^2+^ cooperatively activated WT cFBP1. *K*_0.5_ and Hill coefficient values for Mg^2+^ were 2.0 ± 0.1 and 1.9 ± 0.1, respectively (Table 1), which is consistent with previous reports on kinetic properties of plant cFBP1s (Charles and Halliwell 1980; Serrato et al. 2009) and mammalian FBPases (Chen et al. 1993; Nelson et al. 2004). In contrast, the C95S cFBP1 variant exhibited *K*_0.5_ and Hill coefficient values for Mg^2+^ of 4.5 ± 0.3 and 1.5 ± 0.1, respectively (Table 1).

**Table 1:** Kinetic constants of recombinant mature WT and C95S cFBP1.

cFBP1	*K*_cat_ (s^*−*1^)	*K*_m_ (µM) FBP	Hill coefficient Mg^2+^	*K*_0.5_ (mM) Mg^2+^
WT	23.5 ± 0.3	68.0 ± 3.0	1.9 ± 0.1	2.0 ± 0.1
C95S	10.7 ± 0.2*	41.0 ± 4.0*	1.5 ± 0.1*	4.5 ± 0.3*

*Note:* Values represent catalytic activity (*K*_cat_), F6P and Mg^2+^ specificity (*K*_m_) and Mg^2+^ cooperativity (Hill coefficient and *K*_0.5_) of three independent experiments. The non‐parametric Mann–Whitney *U*‐test was applied to evaluate statistical differences between enzymatic parameters.

* Denotes statistically significant differences at *p* < 0.05.

### 3.3 C95S cFBP1 Expression Under the Control of *promcFBP1* Only Partially Restores to WT Levels the Reduced Photosynthetic Activities of *cfbp1* Plants

**Figure 3:**
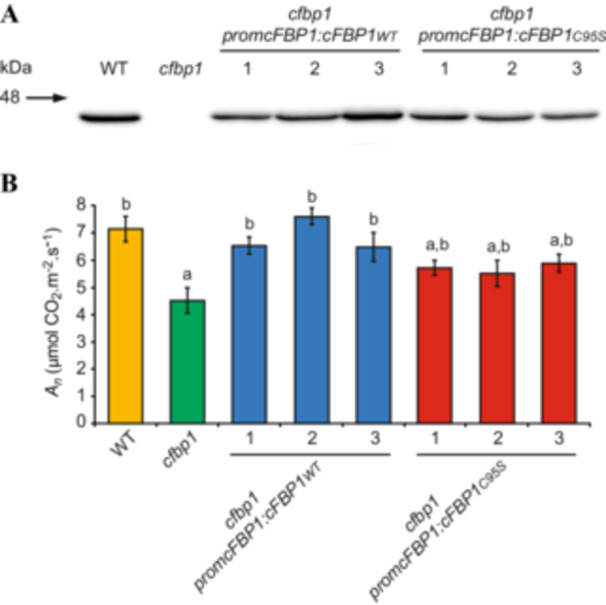
C95S cFBP1 expression under the control of promcFBP1 only partially reverts to WT the reduced photosynthetic activity of *cfbp1* plants. (A) Western blot of cFBP1 and (B) net CO_2_ assimilation rates (*A_n_*) at 400 ppm CO_2_ in leaves of WT and *cfbp1* plants and three independent lines each (1–3) of WT and C95S cFBP1‐expressing *cfbp1* plants. In (A), the gel was loaded with 60 μg of protein per lane. In (B), values are means ± SE determined from three independent experiments using eight plants in each experiment. Lowercase letters indicate significant differences, according to Student's *t*‐test (*p* < 0.05), between: ‘a’ WT and mutant plants and ‘b’ *cfbp1* plants and WT and WT and C95S cFBP1‐expressing *cfbp1* plants. [Color figure can be viewed at wileyonlinelibrary.com]

To investigate the possible dependence of photosynthesis on the Cys95 residue of cFBP1, we measured net rates of photosynthetic CO_2_ assimilation (*A*_*n*_) of WT, *cfbp1* and *cfbp1* plants transformed with *promcFBP1:cFBP1*_*WT*_ and *promcFBP1:cFBP1*_*C95S*_, which expressed the WT and C95S cFBP1 forms under the control of the 1094 bp *promcFBP1* region immediately upstream the translation start codon of *cFBP1*, respectively. The rationale behind this experimental approach was that, if Cys95 is important for cFBP1 activity, *A*_*n*_ values of WT plants and *cfbp1* plants expressing the WT form of cFBP1 should be higher than those of *cfbp1* plants expressing the C95S cFBP1 variant. Conversely, if Cys95 is not a major determinant of cFBP1 activity, C95S cFBP1‐expressing *cfbp1* plants should have WT *A*_*n*_ values. We characterised three independent lines, each of WT and C95S cFBP1‐expressing *cfbp1* plants. Expression of the recombinant proteins was confirmed by western blot analyses (Figure 3A). In keeping with Rojas‐González et al. (2015), *cfbp1* leaves had reduced *A*_*n*_ (60% of WT levels) (Figure 3B). As expected, WT cFBP1 expression countered the reduced *A*_*n*_ values of *cfbp1* plants, reverting them to the WT (Figure 3B). In contrast, C95S cFBP1 expression only slightly enhanced the photosynthetic activities of *cfbp1* plants, as *A*_*n*_ values in *cfbp1* leaves transformed with *promcFBP1:cFBP1*_*C95S*_ were ca. 75% of those of WT leaves, respectively (Figure 3B).

### 3.4 C95S cFBP1 Expression Is Sufficient to Revert the Dwarf Growth Phenotype of *cfpb1* Plants Back to That of WT

**Figure 4:**
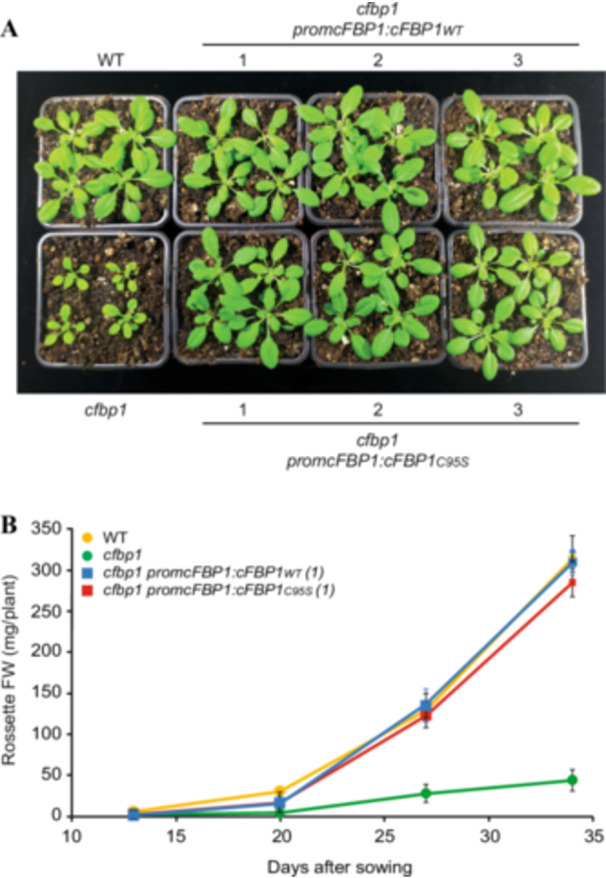
C95S cFBP1 expression is sufficient to revert the dwarf growth phenotype of *cfpb1* plants back to that of WT. (A) External phenotype of WT and *cfbp1* plants, and one representative line each of WT and C95S cFBP1‐expressing *cfbp1* plants. (B) Time series for fresh weight (FW) of rosettes of WT and *cfbp1* plants, and one representative line each of WT and C95S cFBP1‐expressing *cfbp1* plants. In (B), values are means ± SE determined from three independent experiments using eight plants in each experiment. [Color figure can be viewed at wileyonlinelibrary.com]

We compared the growth rates of WT and *cfbp1* plants and *cfbp1* plants expressing WT cFBP1 or C95S cFBP1 under the control of *promcFBP1*. In keeping with Rojas‐González et al. (2015), *cfbp1* plants showed a slow growth phenotype when compared with that of WT plants (Figure 4). Furthermore, the expression of the WT and C95S cFBP1 forms reverted to WT the dwarf phenotype of *cfbp1* plants.

### 3.5 C95S cFBP1 Expression Only Partially Restores to WT the Weak Photosynthetic Response of *cfbp1* Plants to Small Microbial VCs

**Figure 5:**
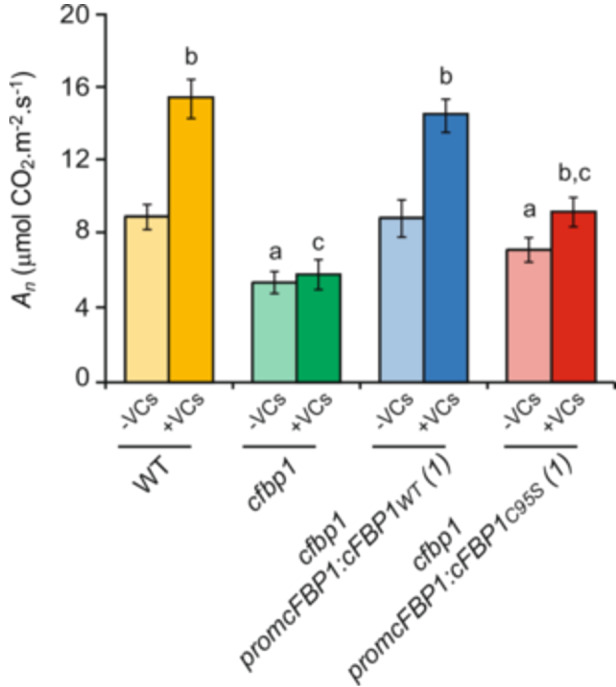
C95S cFBP1 expression only partially restores to WT the weak photosynthetic response of *cfbp1* plants to small microbial VCs. Net CO_2_ assimilation rates (*A_n_*) at 400 ppm CO_2_ in leaves of WT and *cfbp1* plants and one representative line each of WT cFBP1‐ and C95S cFBP1‐expressing *cfbp1* plants grown in the absence or continuous presence of small fungal VCs for 1 week. Values are means ± SE determined from three independent experiments using 12 plants in each experiment. Lowercase letters indicate significant differences, according to Student's *t*‐test (*p* < 0.05) between: ‘a’ VC non‐treated WT plants and mutant plants, ‘b’ VC‐treated and non‐treated plants, and ‘c’ VC‐treated WT and mutant plants.

Whether the Cys95 residue of cFBP1 can determine the photosynthetic response of plants to small microbial VCs was investigated by comparing *A*_*n*_ values of WT and *cfbp1* plants, and *cfbp1* plants expressing WT or C95S cFBP1 grown in the absence or presence of small VCs emitted by the fungal phytopathogen *Alternaria alternata*. In keeping with Ameztoy et al. (2021), *cfbp1* plants had lower *A*_*n*_ values than WT plants when grown in the absence of small fungal VCs (Figure 5). Unlike in WT plants and *cfbp1* plants expressing WT cFBP1 under the *promcFBP1* control, small VCs did not stimulate photosynthesis in *cfbp1* plants (Figure 5), which is consistent with Ameztoy et al. (2021). Increases of *A*_*n*_ values promoted by VCs in C95S cFBP1‐expressing *cfbp1* plants were weaker than in WT cFBP1‐expressing *cfbp1* plants (Figure 5), strongly indicating that Cys95 mediates the photosynthetic response of plants to fungal VCs.

### 3.6 C95S cFBP1 Expression Is Sufficient to Totally Revert the Weak Growth Response to Microbial VCs of *cfpb1* Plants Back to That of WT

We compared the growth responses of WT and *cfbp1* plants and *cfbp1* plants expressing WT cFBP1 or C95S cFBP1 to VCs emitted by *A. alternata*. In keeping with Ameztoy et al. (2021), fungal VCs weakly promoted the growth of *cfbp1* plants (Figure S3). Furthermore, fungal VCs promoted a growth enhancement similar to WT in *cfbp1* plants expressing the WT and C95S cFBP1 forms under the *promcFBP1* control.

## 4 Discussion

### 4.1 Active cFBP1 Is Strictly Dimeric at pH Values Occurring in the Stroma of Illuminated Chloroplasts

Chloroplast pH homoeostasis plays an important role in regulating photosynthesis and related enzymes (Trinh and Masuda 2022). Upon exposure to light, the pH of the chloroplast stroma increases from ca. 7.0 occurring in darkness to 8.0–8.5. Results presented in Figure 1 provide strong evidence that active cFBP1 is strictly dimeric at pH values occurring in the stroma of illuminated chloroplasts, but inactive and tetrameric under neutral pH conditions occurring in the stroma of chloroplasts in the dark. We thus propose that cFBP1 activity and photosynthesis are regulated, at least in part, by stromal pH changes‐driven transitions between inactive tetrameric cFBP1 and active dimeric cFBP1 forms. This hypothesis is consistent with Ocón et al. (2001), who showed that the formation of the regulatory Trx *f‐*cFBP1 complex was better at pH 7.9 than at neutral pH. This undermines the traditionally held view that, similarly to mammalian FBPases (Ke et al. 1990; Xue et al. 1994), active cFBP1 is a strictly tetrameric enzyme that is not subject to regulation by pH‐dependent conformational changes (Villeret et al. 1995; Rodríguez‐Suárez et al. 1997; Chiadmi 1999). This idea is based on the crystallographic studies of Villeret et al. (1995), Chiadmi (1999) and Gütle et al. (2016), who showed that reduced spinach cFBP1 and oxidised cFBP1 from pea and the moss *Physcomitrella patents* adopted a tetrameric quaternary structure. However, these studies were conducted using crystals grown in pH 5.0–5.5 solutions in which cFBP1 is totally inactive. The view that active cFBP1 is a strictly tetrameric enzyme is also based on the fact that, at pH 8.8, cFBP1 has a dimeric structure almost devoid of activity (Zimmermann et al. 1976; Buc et al. 1980; Ballicora and Wolosiuk 1990). However, as shown in this study, dimeric cFBP1 is fully active at pH values ranging between 8.0 and 8.5, and much less active at pH 8.8 (Figure 1C). That dimers of recombinant cFBP1 are active (Figure 1) is consistent with Wiśniewski et al. (2017) who, using recombinant dimeric variants of muscle FBPase with disrupted tetramerisation capacities, demonstrated that dimers of FBPase are also active in solutions and play important roles in multiple processes in vivo (see also Duda et al. 2020, 2021). These authors proposed that, contrary to the well‐established idea that FBPase activity regulation involves a switch between T and R states of tetramers through mechanisms of rotation of the upper and lower dimers (Ke et al. 1990; Xue et al. 1994), FBPase activity allosteric regulation involves dimers exchange between tetramers. We must emphasise that discrepancies between the roles played by single Cys residues in the structural properties of highly similar animal and plant enzymes are not restricted to FBPase. For instance, despite the overall similarity, active animal UDPglucose pyrophosphorylases are strictly octomeric, whereas those of plants exist as a mixture of polymers, dimers and active monomers whose balance is subject to regulation by the redox status of single Cys residues (Martz et al. 2002; Decker et al. 2023).

### 4.2 Cys95 Is an Important Structural Determinant of cFBP1 at pH Values Occurring In the Stroma of Chloroplasts Under Dark and Light Conditions

Cys residues are highly conserved at functionally important sites on proteins (Marino and Gladyshev 2010). A previous site‐directed mutagenesis study on the structural and functional roles of the seven highly conserved Cys residues of cFBP1 showed that Cys96 of rapeseed cFBP1 (Cys 95 in Arabidopsis) is not important for the regulation, structure and catalytic activity of the enzyme (Rodríguez‐Suárez et al. 1997). However, the results presented here provided strong evidence for the other way around. First, replacement of the Cys95 residue by Ser strongly reduced the activity of cFBP1 (Figures 1 and 2) and its Mg^2+^ binding affinity and cooperativity (Table 1, Figure 2). Second, in fresh preparations of recombinant cFBP1, the WT form was mainly present as active dimers at pH 7.8, whereas the C95S variant was mainly present as inactive tetramers (Figure 1E, left panel). This indicates that the Cys95 residue is an important determinant of pH‐dependent cFBP1 oligomerisation status and thus, activity. Third, C95S cFBP1 expression only partially reverted the reduced photosynthetic activity of *cfbp1* plants back to the WT (Figure 3B). Fourth, C95S cFBP1 expression only partially restored to WT the weak photosynthetic response of *cfbp1* plants to small microbial VCs (Figure 5). Our finding that Cys95 plays an important role in the structure and activity of *A. thaliana* cFBP1 is consistent with recent quantitative proteomics analyses showing that changes of the *A. thaliana* cysteine thiol redox proteome occurring under fluctuating light are associated with switches in the redox status of cysteine residues of photosynthesis‐related proteins, including the Cys95 of cFBP1 (Chen et al. 2022). That WT cFBP1 inactivates over time of storage, reaching values comparable to those of C95S cFBP1 (Figure 1B), likely explains the discrepancies between our results on the functional roles of Cys95 and those of Rodríguez‐Suárez et al. (1997).

WT cFBP1 dimers are more active than those of C95S cFBP1 (Figure 1E). This, and the facts that (i) *K*_m_ values of recombinant WT cFBP1 and C95S cFBP1 for FBP were significantly different (Table 1), and (ii) WT cFBP1 and C95S cFBP1 exhibited different cooperativity with respect to Mg^2+^ (Table 1), provided strong evidence that Cys95 has both structural and catalytic roles and mediates in substrate binding to the enzyme. In the Arabidopsis mature cFBP1, the residues which define the Mg^2+^ binding domain are Glu108, Asp129 and Asp132 (Figures S1and S4), as deduced from crystallographic and site‐directed mutagenesis studies of tetrameric forms of pig FBPase (Chen et al. 1993; Nelson et al. 2004) and cFBP1 of pea and spinach (Villeret et al. 1995; Chiadmi 1999). From the same studies it can be inferred that the residues which define the active FBP binding site of Arabidopsis mature cFBP1 monomer are Asp132, Gly133, Asn238, Arg269, Tyr270, Tyr290, Lys300 and Arg302, which are located near the interface between two monomers (Villeret et al. 1995; Chiadmi 1999) (Figure S1). Notably, artificial intelligence‐based AlphaFold protein structure analyses predicted that replacement of Cys95 by Ser or Ala promotes conformational changes in the tetrameric cFBP1 that affect the relative positions of amino acids around the Mg^2+^‐ and FBP‐binding domains (Figure S4A) and Mg^2+^ ions (Figure S4B), thus providing further evidence that Cys95 is an important determinant for the FBP and Mg^2+^ accessibility to the enzyme.

**Figure 6:**
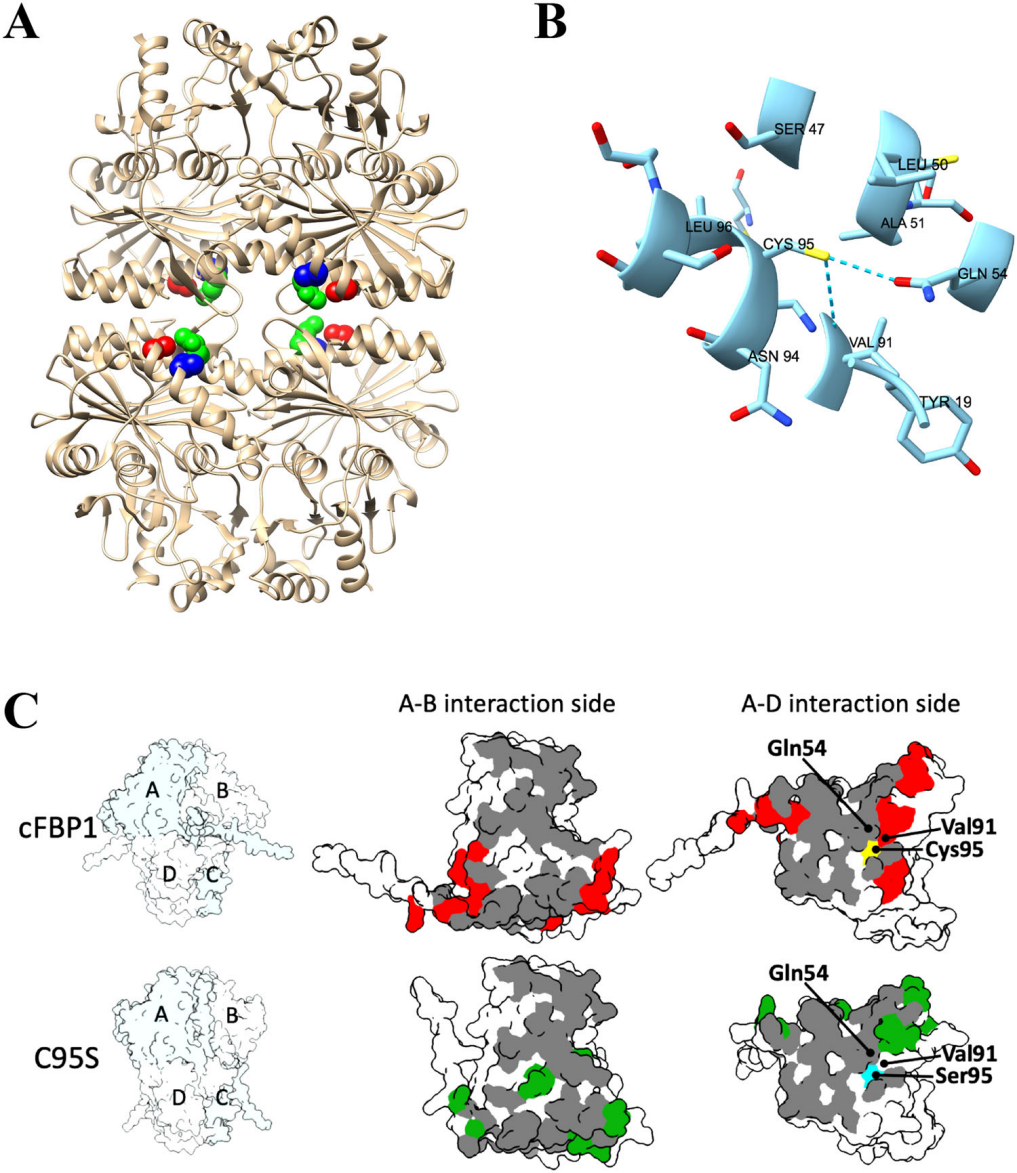
(A) Crystal structure of the pea cFBP1 tetramer available in the Protein Data Bank (PDB code 1D9Q) and locations of the Cys92, Gln51 and Val87 residues (Cys95, Gln54 and Val91 in Arabidopsis). Cys92, Gln51 and Val87 residues are highlighted in red, green and blue, respectively. (B) AlphaFold predicted the formation of hydrogen bridges between Cys95 and Gln54 and Cys95 and Val91 of Arabidopsis cFBP1. (C) AlphaFold predicted surface view of Arabidopsis WT and C95S cFBP1. On the left, the overall quaternary structure of WT cFBP1 and C95S cFBP1, with subunits shown in blue (A and C) and white (B and D). The residues that are involved in subunit interactions in both WT cFBP1 and C95S cFBP1 are indicated in grey, while those that are specific to each form are shown in red and green, respectively. [Color figure can be viewed at wileyonlinelibrary.com]

Free Cys is highly nucleophilic due to the large atomic radius of its sulphur atom. Its nucleophilicity is governed by the pKa and ionisation state of the thiol, which in turn depend upon the interaction with the local microenvironment (Bulaj et al. 1998). Although Cys residues in proteins are often buried (Marino and Gladyshev 2010), our analyses of crystallographic data of pea and spinach cFBP1 available on the RCSB Protein Data Bank (https://www.rcsb.org/structure/1D9Q#entity-1; https://www.rcsb.org/structure/1SPI#entity-1) revealed that Cys95 is located at the surface of the dimer (Figure 6A). The predicted pKa for a surface‐exposed Cys is close to 8.0 (Marino and Gladyshev 2010; Bak et al. 2019). Therefore, ionisation and reactivity of Cys95 of cFBP1 would be prone to regulation by pH oscillations that occur in the stroma of chloroplasts under light and dark conditions. Cys can stabilise α helices using weak hydrogen bonds (Mazmanian et al. 2016). Our analyses using AlphaFold predicted that Cys95 can form hydrogen bridges with Gln54 and Val91 (Figure 6B), two surface‐exposed amino acids located in α‐helices that are involved in the interactions between dimers. Consistently, the same analyses predicted that replacement of Cys95 by Ser or Ala alters the sets of amino acids involved in the interactions between dimers (Figure 6C). Thus, Arg14, Ser17, Glu20, Leu21, Leu58, Arg61, Ile87, Val91, Arg97 and Glu118, which are predicted by AlphaFold to be involved in the interaction between WT cFBP1 dimers, are not predicted to be involved in the interaction between C95S cFBP1 dimers. Moreover, Gly18, Ala73, Val74, Arg61, Gly72, Ala73 and Asn75, which are predicted to be involved in interactions of C95S cFBP1 dimers, are not predicted to be involved in the interaction between WT cFBP1 dimers. Therefore, it is highly conceivable that Cys95 is an important determinant of the pH‐driven structure, activity and regulatory properties of cFBP1. Further three‐dimensional structural analyses of WT and C95S cFBP1 crystals grown under both neutral and alkaline conditions will be necessary to better understand how the Cys95 residue mediates the pH‐dependent conformational status of cFBP1.

### 4.3 Individually, Cys95 of the ‘Excess’ Enzyme cFBP1 Is an Important Determinant of Photosynthesis, but Not of Growth in Arabidopsis

Some enzymes of the CBC, including cFBP1, phosphoribulokinase and glyceraldehyde‐3‐phosphate dehydrogenase, have reduced flux‐control over CO_2_ assimilation and growth (Koßmann et al. 1994; Paul et al. 1995; Price et al. 1995). Previous studies using cFBP1 antisense potato plants showed that the plastidial FBPase activity can be reduced by 85% before an effect on growth is observed, strongly indicating that cFBP1 is present in excess (Koßmann et al. 1994). The same studies showed that a 36% reduction in plastid FBPase activity resulted in a 20% reduction in photosynthetic activity, but still, plants exhibited a WT growth phenotype. *cfbp1* plants have ca. 60% of the WT photosynthetic activity and a slow growth phenotype (Rojas‐González et al. 2015) (Figures 3B and 4, this study). Here we found that, despite having 75%–80% of the WT photosynthetic activities, C95S cFBP1‐expressing *cfbp1* plants did not exhibit growth retardation under both on soil and in vitro growth conditions (Figures 3B and 4). It thus appears that (i) Cys95 is an important mediator in both cFBP1 activity and photosynthesis in *Arabidopsis*, (ii) the maximal photosynthetic activity in C95S cFBP1 expressing *cfbp1* plants is greater than that required for full growth under laboratory conditions and (iii) cFBP1 is present in excess in WT Arabidopsis plants. Furthermore, we found that, despite promoting lower than WT increases in photosynthetic activity, the microbial VC treatment promoted a growth increase similar to WT in C95S cFBP1 expressing *cfbp1* plants (Figures 5 and S3). This strongly indicated that (i) Cys95 is an important determinant of the photosynthetic response to fungal VCs; (ii) individually, changes in the redox state of the Cys95 residue of cFBP1 in VC‐exposed plants exert a minor control, if any, on plant growth; (iii) the combined action of factors other than Cys95 reduction are important for microbial VC‐promoted growth, and (iv) photosynthesis is not the main rate‐controlling factor for growth in VC‐exposed plants.

### 4.4 Additional Remarks

Regulation of photosynthesis by light involves Fdx/Trx *f* and NTRC‐mediated reduction of disulphide bridges between Cys residues of photosynthesis‐related proteins (Nikkanen et al. 2016; Cejudo et al. 2021; Yoshida and Hisabori 2023). Environmental changes, including microbial VC exposure and fluctuating light, promote reversible changes in the redox status of Cys95 of cFBP1 and other highly conserved Cys residues of photosynthesis‐related proteins (Ameztoy et al. 2019; Chen et al. 2022). These changes do not involve the reversible formation of disulphide bridges. Reversible redox thiol redox modifications of Cys, such as S‐glutathionylation, S‐nitrosylation and oxidation to sulfenic acid, provide posttranslational ‘switches’ in many regulatory mechanisms that allow plants to adjust to changing environmental constraints (Couturier et al. 2013; Cannon and Horn 2023). Here we showed that Cys95 of Arabidopsis cFBP1 is an important determinant of the pH‐driven structure and activity of the enzyme. Therefore, we propose that changes in the redox status of Cys95 of cFBP1 and of other highly conserved Cys residues of photosynthesis‐related proteins that do not form disulphide bridges would represent a second important mechanism for regulation of photosynthesis, especially in response to changes in the environmental factors that give rise to oxidative stress and changes in the chloroplastic pH.

## Supporting information

Supplemental Figure S1.

Supplemental Figure S2.

Supplemental Figure S3.

Supplemental Figure S4.

Supplemental Table S1 ff(1).

Supmat.

## Data Availability

The data that support the findings of this study are available on request from the corresponding author. The data are not publicly available due to privacy or ethical restrictions.
