# Supplementary material for: The Highly Conserved Cys95 Residue of Fructose‐1,6‐Bisphosphatase 1 Mediates the pH‐Driven Structure and Activity of the Enzyme and Photosynthesis
Source: Plant Cell Environ. 2025 Jun 8;48(9):6941–51. doi: 10.1111/pce.15667 (PMC12319266; doi:10.1111/pce.15667)
Supplement: Supplementary file 1 — Supplemental Figure S1. [file PCE-48-6941-s003.pdf]

```
P. sativum cFBP1            ----MAVKEATSETKKRSGYEIITLTSWLLQQE-QKGIIDAELTIVLSSISMACKQIASL     55
S. oleracea cFBP1           --AAVGEAATETKARTRSKYEIETLTGWLLKQE-MAGVIDAELTIVLSSISLACKQIASL     57
A. thaliana cFBP1           -AVAADAAETKTAARKKSGYELQTLTGWLLRQE-MKGEIDAELTIVMSSISLACKQIASL     58
B. napus cFBP1              AVAADATAETKPAAKKKSGYELQTLTSWLLRQE-MKGEIDTELTIVMSSIAMACKQIASL     59
S. scrofa FBPase            ------------TDQAAFDTNIVTLTRFVMEQG-RKARGTGEMTQLLNSLCTAVKAISTA     47
A. thaliana cytFBPase       ----------MDHAADAHRTDLMTITRFVLNEQSKYPESRGDFTILLSHIVLGCKFVCSA     50
                                     :: *:* :::.:         ::* ::. :  . * :.:

P. sativum cFBP1            VQRANISNLTGTQGAVNIQGEDQKKLDVISNEVFSNCLRSSGRTGIIASEEEDVAVAVEE     115
S. oleracea cFBP1           VQRAGISNLTGIQGAVNIQGEDQKKLDVVSNEVFSSCLRSSGRTGIIASEEEDVPVAVEE     117
A. thaliana cFBP1           VQRAGISNLTGVQGAVNIQGEDQKKLDVISNEVFSNCLRSSGRTGIIASEEEDVPVAVEE     118
B. napus cFBP1              VQRAGISNLTGVQGAVNIQGEDQKKLDVVSNEVFSNCLRSSGRTGIIASEEEDVPVAVEE     119
S. scrofa FBPase            VRKAGIAHLYGIAGATNVTGDQVKKLDVLSNDLVINVLKSSFATCVLVTEEDKNAIIVEP     107
A. thaliana cytFBPase       VNKAGLAKLIGLAGETNIQGEEQKKLDVLSNDVFVNALVSSGRTSVLVSEEDEEATFVEP     110
                            *.:*.:::* *  * .*: *:: *****:**::. . * **  * ::.:**:.    **

P. sativum cFBP1            SYSGNYIVVFDPLDGSSNLDAAVSTGSIFGIYSPNDECLPDFGDDSDDNTLGTEEQRCIV     175
S. oleracea cFBP1           SYSGNYIVVFDPLDGSSNIDAAVSTGSIFGIYSPNDECIVDSDH-DDESQLSAEEQRCVV     176
A. thaliana cFBP1           SYSGNYVVVFDPLDGSSNIDAAVSTGSIFGIYSPNDECIVDD-S-DDISALGSEEQRCIV     176
B. napus cFBP1              SYSGNYVVVFDPLDGSSNIDAAVSTGSIFGIYSPNDECLPD--S-DDTSALGSEEERCIV     176
S. scrofa FBPase            EKRGKYVVCFDPLDGSSNIDCLVSIGTIFGIYRKNSTDEP-----------------SEK     150
A. thaliana cytFBPase       SKRGKYCVVFDPLDGSSNIDCGVSIGTIFGIYTLDHTDEP-----------------TTA     153
                            .  *:* * *********:*. ** *:*****  :

P. sativum cFBP1            NVCQPGSNLLAAGYCMYSSSVAFVLTIGKGVFVFTLDPLYGEFVLTQENLQIPKSGEIYS     235
S. oleracea cFBP1           NVCQPGDNLLAAGYCMYSSSVIFVLTIGKGVYAFTLDPMYGEFVLTSEKIQIPKAGKIYS     236
A. thaliana cFBP1           NVCQPGNNLLAAGYCMYSSSVIFVLTLGKGVFSFTLDPMYGEFVLTQENIEIPKAGRIYS     236
B. napus cFBP1              NVCQPGNNLLAAGYCMYSSSVIFVLTLGKGVFAFTLDPMYGEFVLTQENIEIPKAGKIYS     236
S. scrofa FBPase            DALQPGRNLVAAGYALYGSATMLVLAMVNGVNCFMLDPAIGEFILVDRNVKIKKKGSIYS     210
A. thaliana cytFBPase       DVLKPGNEMVAAGYCMYGSSCMLVLSTGTGVHGFTLDPSLGEFILTHPDIKIPNKGNIYS     213
                            :. :** :::****.:*.*:  :**:  .**  * ***  ***:*.  .::* : * ***

P. sativum cFBP1            FNEGNYKLWDENLKKYIDDLKEPGPSGKPYSARYIGSLVGDFHRTLLYGGIYGYPRDKKS     295
S. oleracea cFBP1           FNEGNYKMWDDKLKKYMDDLKEPGESQKPYSSRYIGSLVGDFHRTLLYGGIYGYPRDAKS     296
A. thaliana cFBP1           FNEGNYQMWDDKLKKYIDDLKDPGPTGKPYSARYIGSLVGDFHRTLLYGGIYGYPRDAKS     296
B. napus cFBP1              FNEGNYQMWDENLKKYIDDLKDPGPSGKPYSARYIGSLVGDFHRTLLYGGIYGYPRDAKS     296
S. scrofa FBPase            INEGYAKEFDPAITEYIQRKKFPPDNSAPYGARYVGSMVADVHRTLVYGGIFMYPANKKS     270
A. thaliana cytFBPase       VNEGNAQNWDGPTTKYVEKCKFPKDGSPAKSLRYVGSMVADVHRTLLYGGIFLYPADKKS     273
                            .***  : :*   .:*::  * *       . **:**:*.*.****:****: ** : **

P. sativum cFBP1            KNGKLRLLYECAPMSFIVEQAGGKGSDGHQRVLDIQPTEIHQRVPLYIGSTEEVEKVEKY     355
S. oleracea cFBP1           KNGKLRLLYECAPMSFIVEQAGGKGSDGHQRILDIQPTEIHQRVPLYIGSVEEVEKLEKY     356
A. thaliana cFBP1           KNGKLRLLYECAPMSFIVEQAGGKGSDGHSRVLDIQPTEIHQRVPLYIGSTEEVEKLEKY     356
B. napus cFBP1              KNGKLRLLYECAPMSFIVEQAGGKGSDGHHRVLDIQPTEIHQRVPLYIGSKEEVEKLEKY     356
S. scrofa FBPase            PKGKLRLLYECNPMAYVMEKAGGLATTGKEAVLDIVPTDIHQRAPIILGSPEDVTELLEI     330
A. thaliana cytFBPase       PNGKLRVLYEVFPMSFLMEQAGGQAFTGKKRALDLVPEKIHERSPIFLGSYDDVEEIKAL     333
                            :****:***  **::::*:*** .  *:   **: * .**:* *: :** ::* ::

P. sativum cFBP1            LA------      357
S. oleracea cFBP1           LA------      358
A. thaliana cFBP1           LA------      358
B. napus cFBP1              LA------      358
S. scrofa FBPase            YQKHA---      335
A. thaliana cytFBPase       YAEEEKKN      341
```

Cysteine

Cysteine-95

FBP binding site

Mg^{2+} binding site

Mg^{2+} and FBP binding site

Supplemental Figure S1: Multiple sequence alignment of the amino acid sequences of mature cFBP1 from pea (*Pisum sativum*) (NCBI accession number AAD10213.1), rapeseed (*Brassica napus*) (NCBI accession number NP_001302992.1), spinach (*Spinacia oleracea*) (NCBI accession number AAD10207.1) and Arabidopsis (*A. thaliana*) (NCBI accession number CAA41154.1), pig (*Sus scrofa*) fructose-1,6-bisphosphatase (FBPase) (NCBI accession number AAA31035.1) and cytosolic FBPase of Arabidopsis (cytFBPase) (NCBI accession number NP_175032.1) using Clustal Omega (http://www.ebi.ac.uk/Tools/msa/clustalo/). The numbers indicate the amino acid positions. Asterisks (*) indicate positions which have a highly conserved residue; Colon (:) indicates residues with strongly similar physiochemical properties; Period (.) indicates residues with weakly similar properties.
