# Supplementary material for: The Highly Conserved Cys95 Residue of Fructose‐1,6‐Bisphosphatase 1 Mediates the pH‐Driven Structure and Activity of the Enzyme and Photosynthesis
Source: Plant Cell Environ. 2025 Jun 8;48(9):6941–51. doi: 10.1111/pce.15667 (PMC12319266; doi:10.1111/pce.15667)
Supplement: Supplementary file 2 — Supplemental Figure S2. [file PCE-48-6941-s005.pdf]

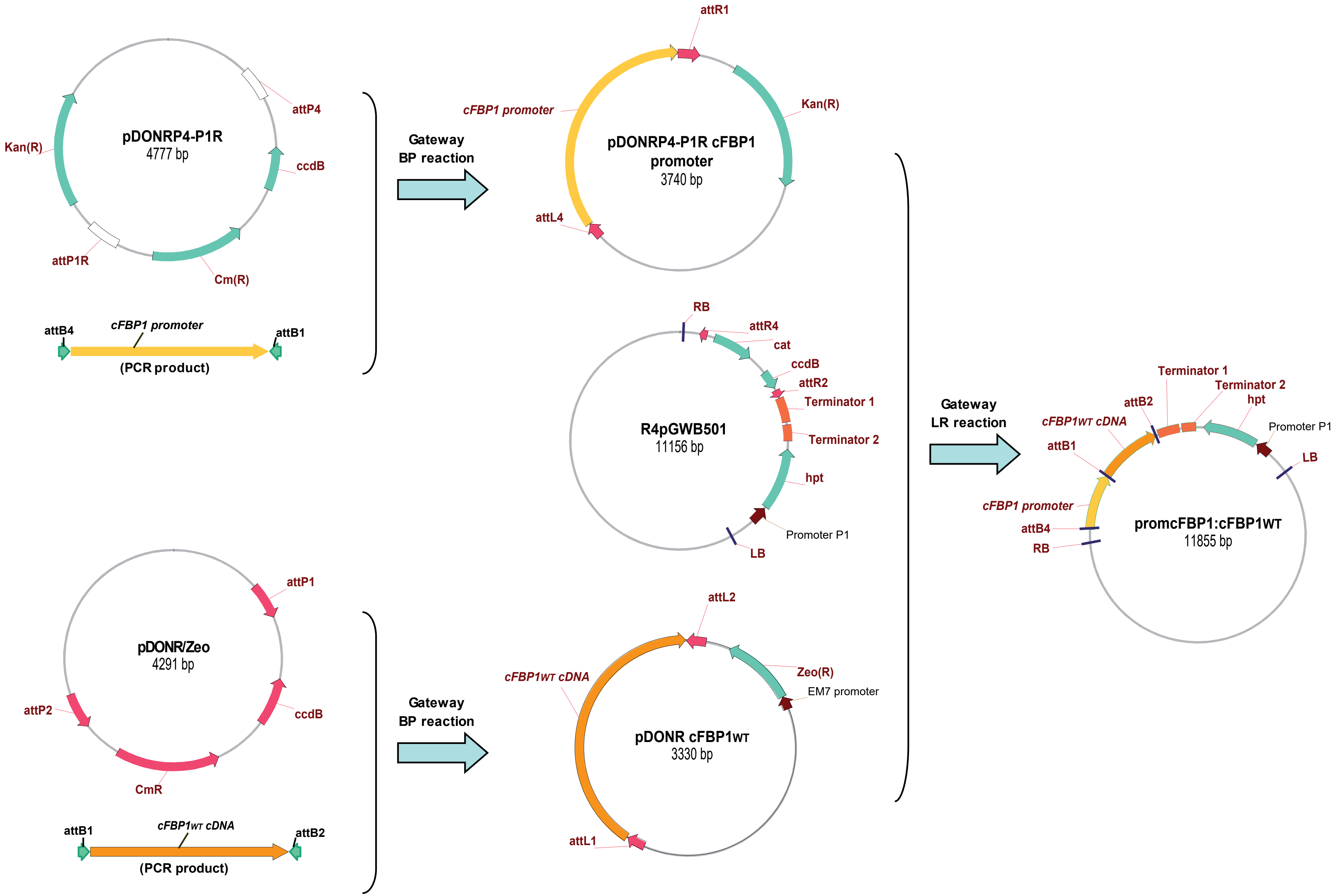

Supplemental Figure S2: Stages in the construction of the promcFBP1:cFBP1WT, promcFBP1:cFBP1C95S, pDEST17- cFBP1WT and pDEST17- cFBP1C95S plasmids. Plasmid constructs were produced using Gateway technology and confirmed by sequencing. Primers used for PCR amplification of complete a cFBP1 cDNA obtained from the RIKEN Arabidopsis cDNA collection (Seki et al., 1998, 2002) are listed in Supplemental Table S1

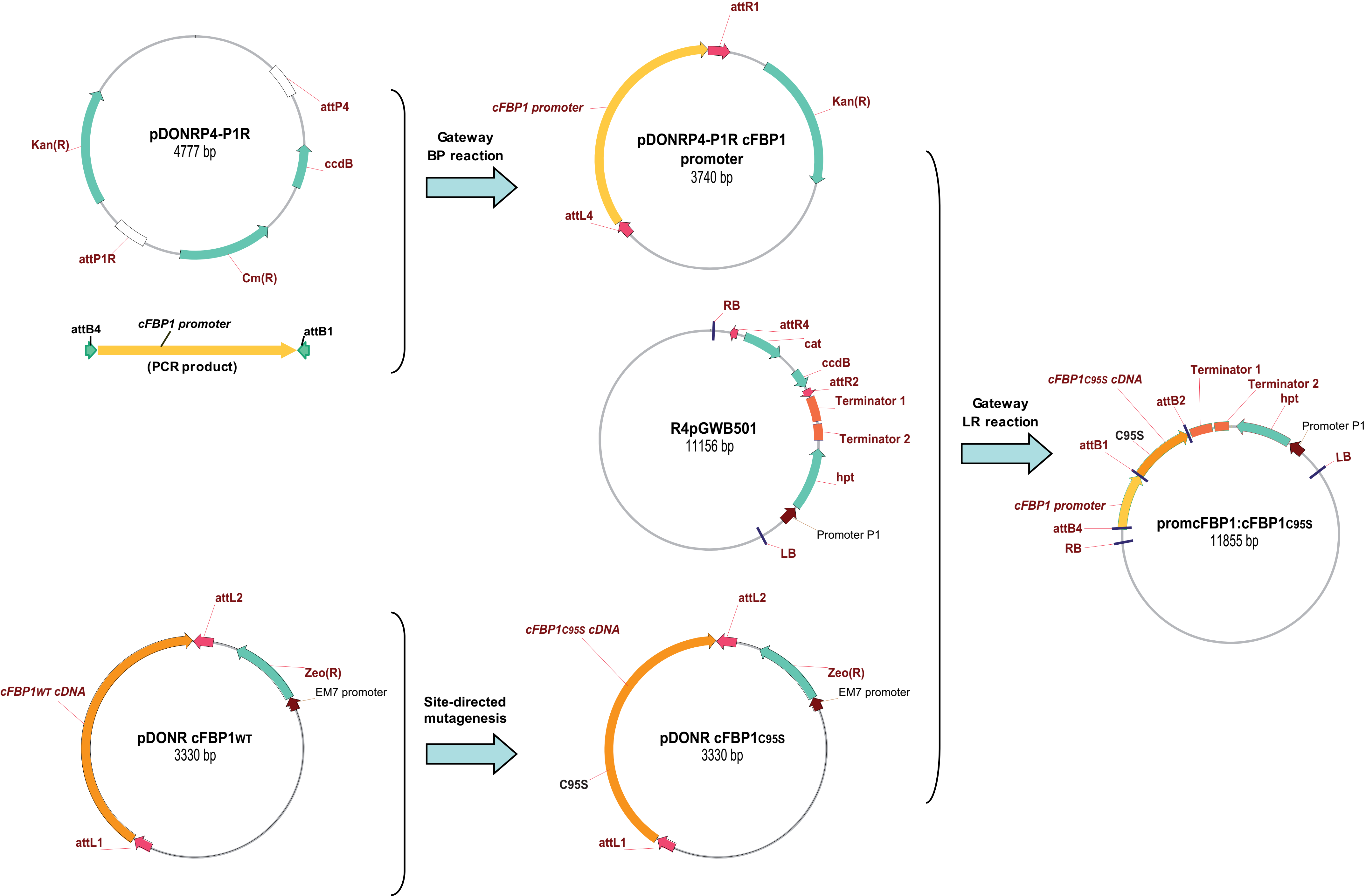
pDONRP4-P1R
4777 bp
attP4
ccdB
Kan(R)
attP1R
Cm(R)
attB4
cFBP1 promoter
attB1
(PCR product)
Gateway
BP reaction
attR1
cFBP1 promoter
Kan(R)
pDONRP4-P1R cFBP1
promoter
3740 bp
attL4
RB
attR4
cat
ccdB
attR2
Terminator 1
Terminator 2
R4pGWB501
11156 bp
hpt
Promoter P1
LB
Gateway
LR reaction
cFBP1C95S cDNA
Terminator 1
Terminator 2
hpt
attB2
Promoter P1
C95S
attB1
LB
cFBP1 promoter
attB4
RB
promcFBP1:cFBP1C95S
11855 bp
attL2
Zeo(R)
cFBP1WT cDNA
EM7 promoter
pDONR cFBP1WT
3330 bp
attL1
Site-directed
mutagenesis
cFBP1C95S cDNA
attL2
Zeo(R)
EM7 promoter
pDONR cFBP1C95S
3330 bp
C95S
attL1

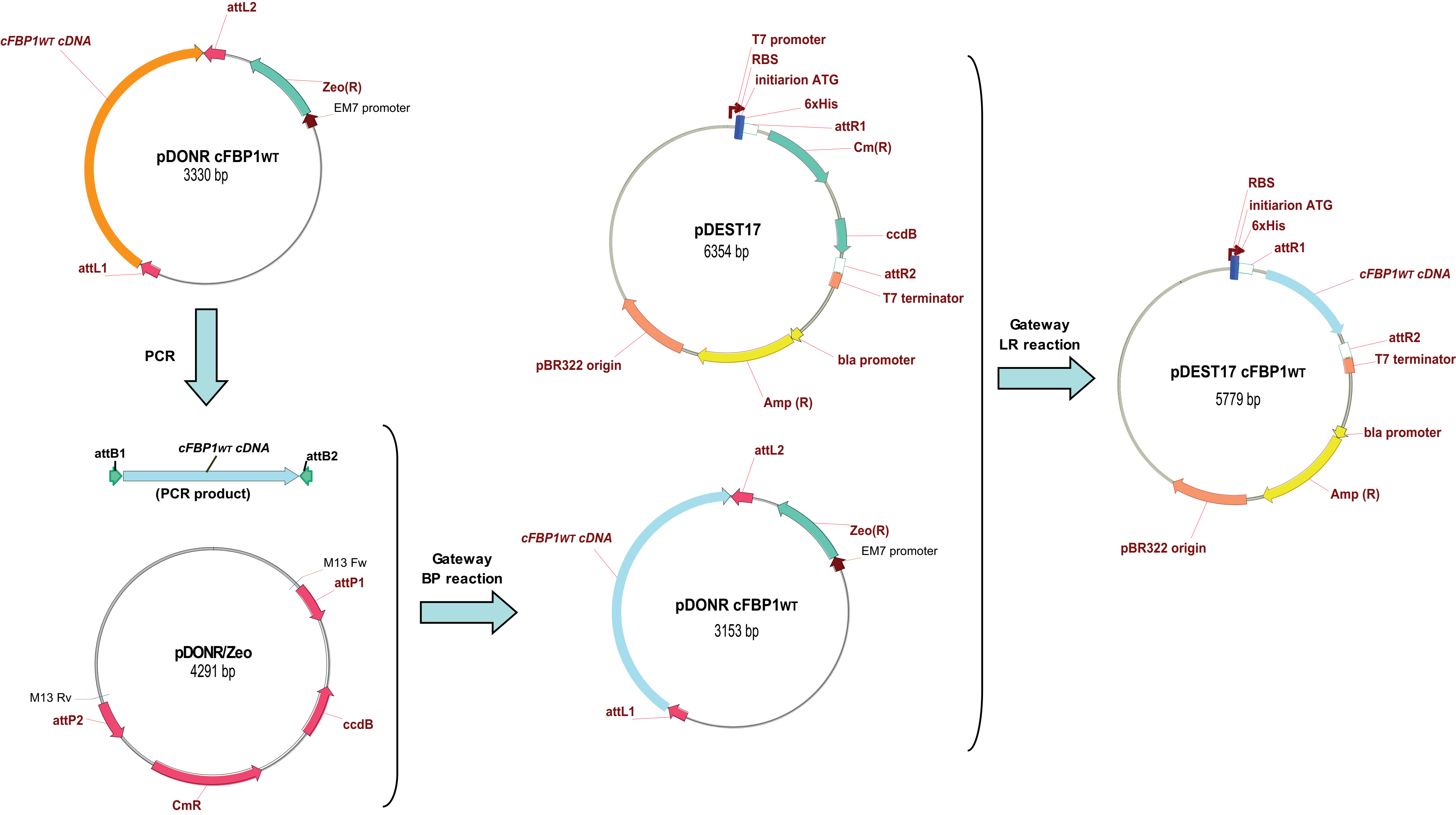
attL2
cFBP1WT cDNA
Zeo(R)
EM7 promoter
pDONR cFBP1WT
3330 bp
attL1
PCR
attB1
cFBP1WT cDNA
attB2
(PCR product)
M13 Fw
attP1
pDONR/Zeo
4291 bp
M13 Rv
attP2
ccdB
CmR
Gateway
BP reaction
T7 promoter
RBS
initiarion ATG
6xHis
attR1
Cm(R)
pDEST17
6354 bp
ccdB
attR2
T7 terminator
bla promoter
pBR322 origin
Amp (R)
attL2
cFBP1WT cDNA
Zeo(R)
EM7 promoter
pDONR cFBP1WT
3153 bp
attL1
Gateway
LR reaction
RBS
initiarion ATG
6xHis
attR1
cFBP1WT cDNA
attR2
T7 terminator
pDEST17 cFBP1WT
5779 bp
bla promoter
Amp (R)
pBR322 origin

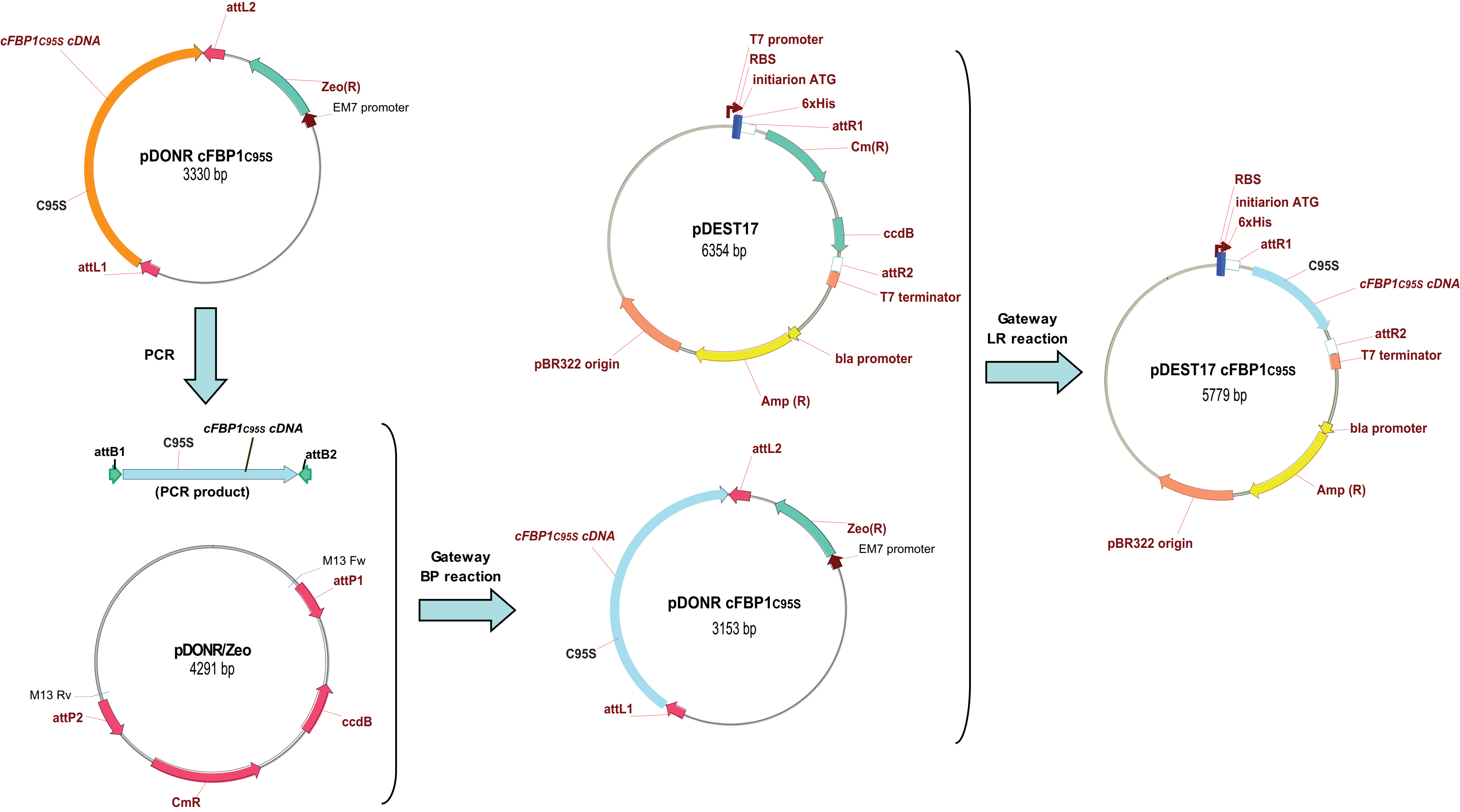
pDONR cFBP1C95S
3330 bp
cFBP1C95S cDNA
attL2
Zeo(R)
EM7 promoter
C95S
attL1
PCR
attB1
C95S
cFBP1C95S cDNA
attB2
(PCR product)
pDONR/Zeo
4291 bp
M13 Fw
attP1
M13 Rv
attP2
ccdB
CmR
Gateway
BP reaction
pDEST17
6354 bp
T7 promoter
RBS
initiarion ATG
6xHis
attR1
Cm(R)
ccdB
attR2
T7 terminator
bla promoter
Amp (R)
pBR322 origin
pDONR cFBP1C95S
3153 bp
attL2
Zeo(R)
EM7 promoter
cFBP1C95S cDNA
C95S
attL1
Gateway
LR reaction
pDEST17 cFBP1C95S
5779 bp
RBS
initiarion ATG
6xHis
attR1
C95S
cFBP1C95S cDNA
attR2
T7 terminator
bla promoter
Amp (R)
pBR322 origin
