# Supplementary material for: The Highly Conserved Cys95 Residue of Fructose‐1,6‐Bisphosphatase 1 Mediates the pH‐Driven Structure and Activity of the Enzyme and Photosynthesis
Source: Plant Cell Environ. 2025 Jun 8;48(9):6941–51. doi: 10.1111/pce.15667 (PMC12319266; doi:10.1111/pce.15667)
Supplement: Supplementary file 3 — Supplemental Figure S3. [file PCE-48-6941-s001.pdf]

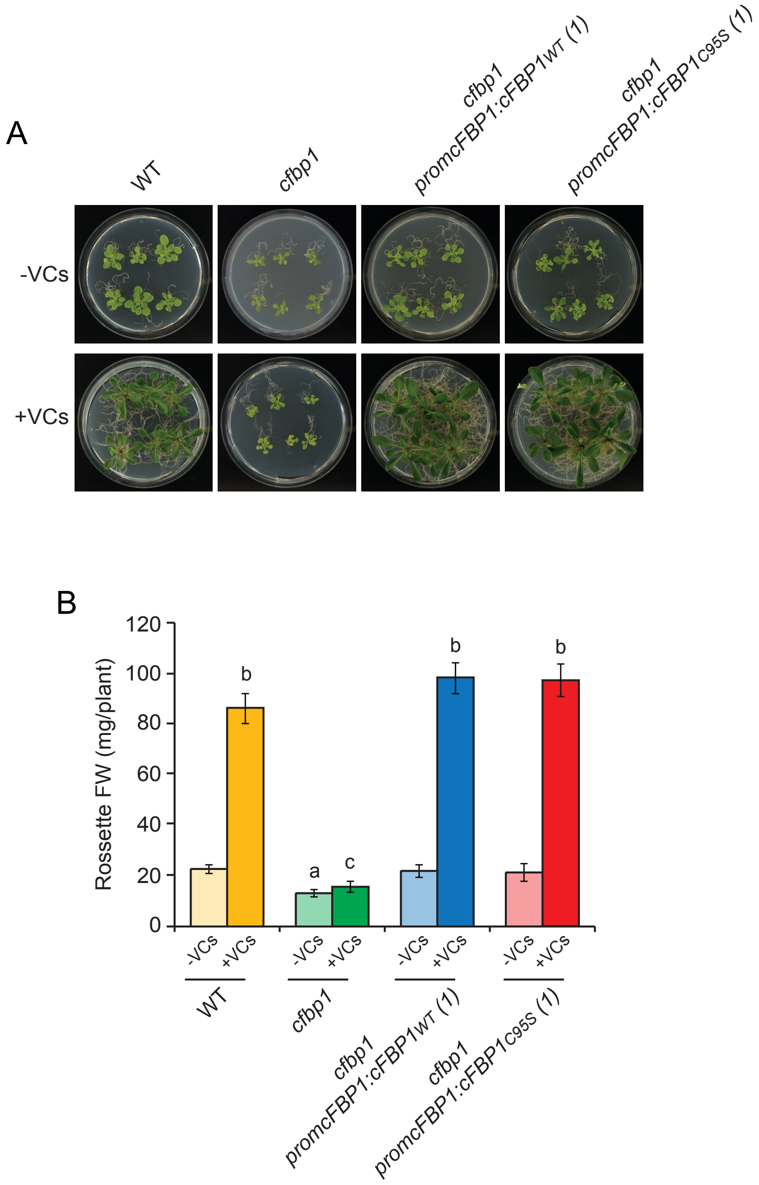

Supplemental Figure S3: C95S cFBP1 expression is sufficient to restore to WT the weak response of *cfbp1* plants to small microbial VCs. (A) External phenotype and (B) rosette fresh weight (FW) of WT and *cfbp1* plants and one representative line each of WT cFBP1- and C95S cFBP1-expressing *cfbp1* plants grown in the absence or continuous presence of small fungal VCs for one week. Values in panel (B) are means ± SE for 3 biological replicates (each a pool of 12 plants) obtained from four independent experiments. In (B), lowercase letters indicate significant differences, according to Student´s t-test (P<0.05) between: "a" VC non-treated WT plants and mutant plants, "b" VC-treated and non-treated plants, and "c" VC-treated WT and mutant plants.
