# Supplementary material for: The Highly Conserved Cys95 Residue of Fructose‐1,6‐Bisphosphatase 1 Mediates the pH‐Driven Structure and Activity of the Enzyme and Photosynthesis
Source: Plant Cell Environ. 2025 Jun 8;48(9):6941–51. doi: 10.1111/pce.15667 (PMC12319266; doi:10.1111/pce.15667)

A

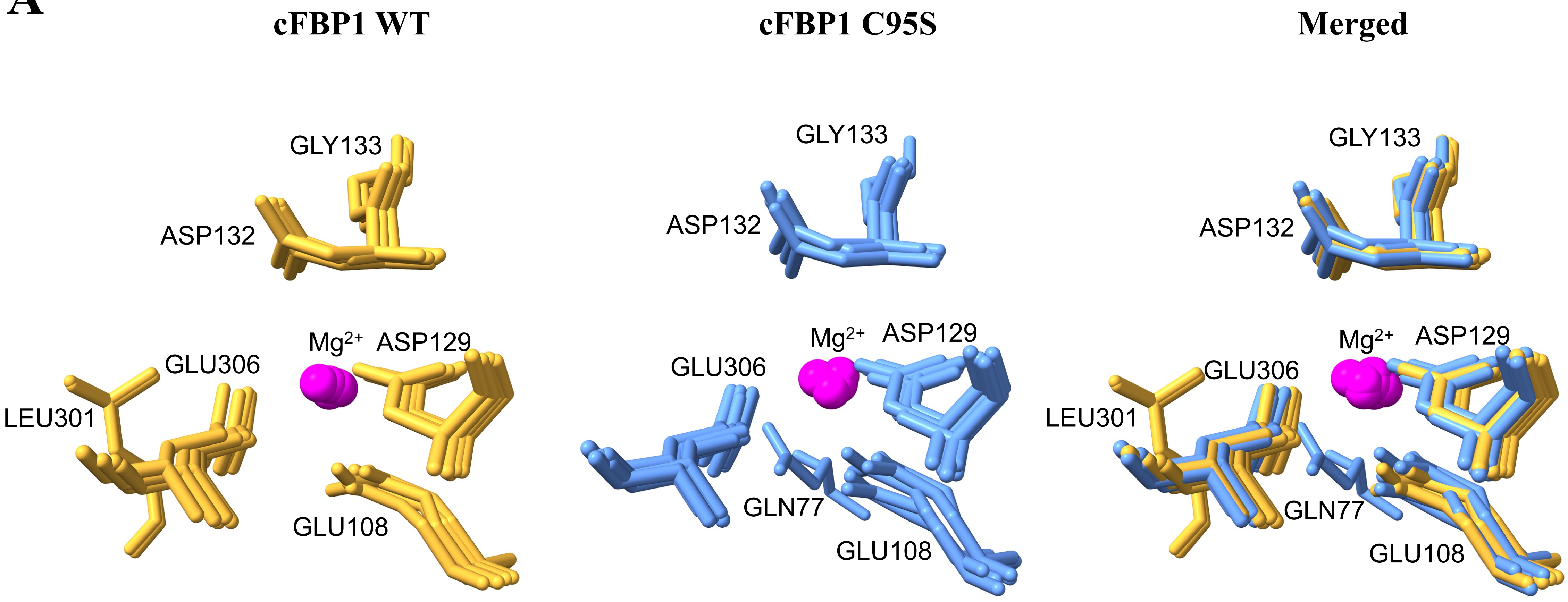

Supplemental Figure S4: AlphaFold predicted conformational changes in the Mg^{2+}- and FBP-binding domains. (A) Amino acids of the Mg^{2+} binding domains located within 5 Å from the Mg^{2+} ion in WT cFBP1 and C95S cFBP1. (B) Mg^{2+}positions in the tetrameric in WT cFBP1 and C95S cFBP1. In (A), the two cFBP1 forms are presented as a ribbon diagrams (each cFBP1 form in a different color, for clarity) and Mg^{2+} ions are shown as pink (A) and green (B) spheres.

B

cFBP1 WT

cFBP1 C95S

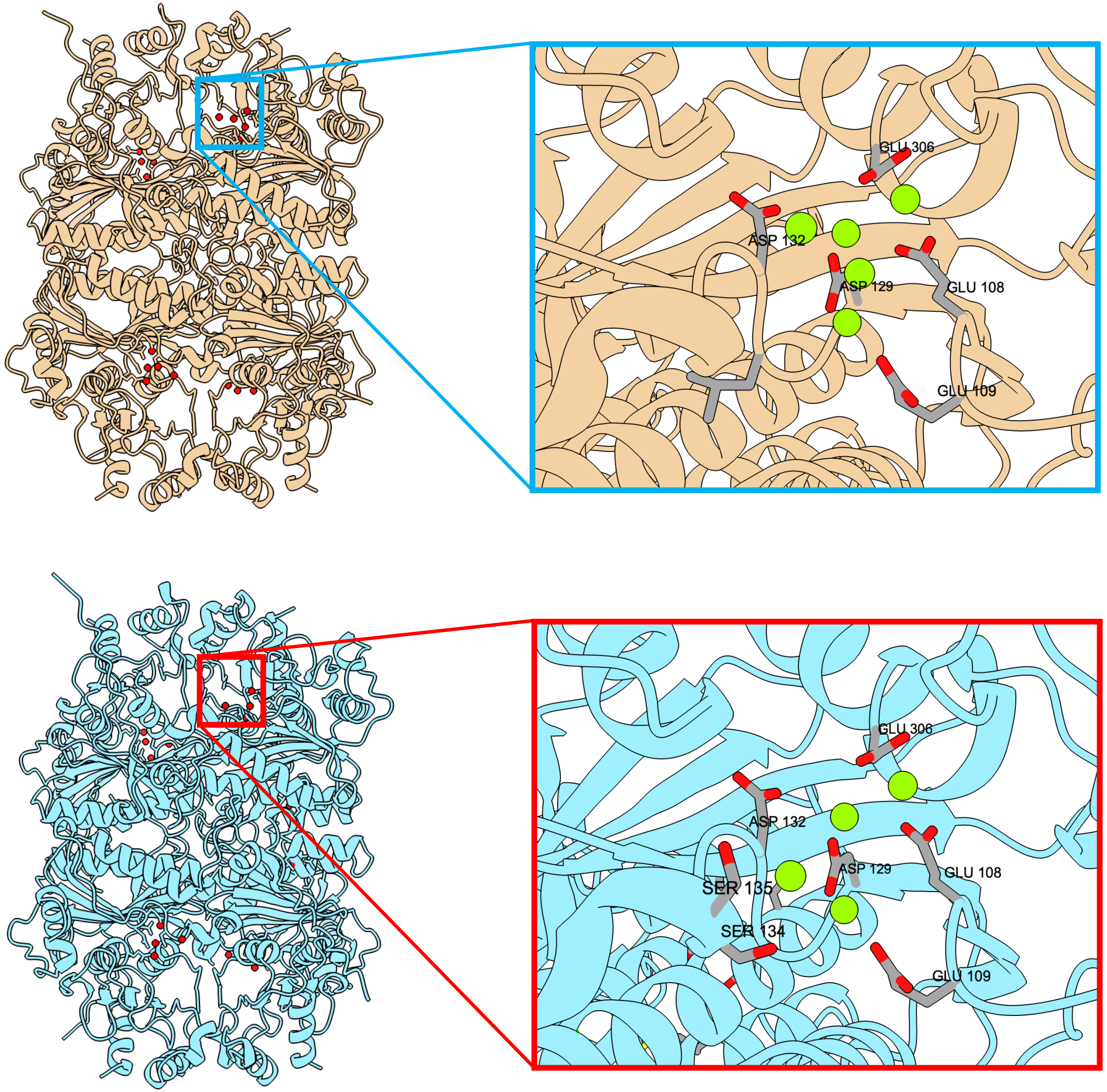

Supplement: Supplementary file 4 — Supplemental Figure S4. [file PCE-48-6941-s002.pdf]
