# Supplementary material for: The Highly Conserved Cys95 Residue of Fructose‐1,6‐Bisphosphatase 1 Mediates the pH‐Driven Structure and Activity of the Enzyme and Photosynthesis
Source: Plant Cell Environ. 2025 Jun 8;48(9):6941–51. doi: 10.1111/pce.15667 (PMC12319266; doi:10.1111/pce.15667)
Supplement: Supplementary file 5 — Supplemental Table S1 ff(1). [file PCE-48-6941-s006.docx]

**Supplemental Table S1.** Primers used for PCR amplification of *cFBP1_WT_*, *cFBP1_C95S_* and *cFBP1* promoter to produce the pDEST17-cFBP1_WT_, pDEST17-cFBP1_C95S_, promcFBP1: cFBP1_WT_ and promcFBP1: cFBP1_C95S_ plasmids. Primer sequences for attB sites (see **Supplemental Figure S2**) are indicated in bold.

| Primer | **Sequence** |
| --- | --- |
| ***cFBP1* for promcFBP1:cFBP1_WT_** |  |
| attB1 *cFBP1* | 5’-**ggggacaagtttgtacaaaaaagcaggctta**atggcagcaaccgccgcaac-3’ |
| attB2 *cFBP1* | 5’-**ggggaccactttgtacaagaaagctgggta**tcaagccaagtacttctccagc-3’ |
|  |  |
| ***cFBP1* for promcFBP1:cFBP1C95S** | |
| *C95S* forward | 5´-ctaatgaggtgttttccaactctttgagatcaagtggaagaac-3’ |
| *C95S* reverse | 5’-gttcttccacttgatctcaaagagttggaaaacacctcattag-3’ |
| ***cFBP1* for pDEST17 cFBP1_WT_ and pDEST17 cFBP1_C95S_** | |
| attB1 *cFBP1** | 5’-**ggggacaagtttgtacaaaaaagcaggctta**gccgtagcggcggatgctg-3’ |
| attB2 *cFBP1* | 5’-**ggggaccactttgtacaagaaagctgggta**tcaagccaagtacttctccagc-3’ |
| ***promcFBP1*** |  |
| attB4 *cFBP1* promoter | 5’-**ggggacaactttgtatagaaaagttgct**ggttaatcaacgattcaatgaactag-3’ |
| attB1 *cFBP1* promoter | 5’-**ggggactgcttttttgtacaaacttgc**tttttctgtgttgttttgaaaaaaac-3’ |
