# Supplementary material for: The Highly Conserved Cys95 Residue of Fructose‐1,6‐Bisphosphatase 1 Mediates the pH‐Driven Structure and Activity of the Enzyme and Photosynthesis
Source: Plant Cell Environ. 2025 Jun 8;48(9):6941–51. doi: 10.1111/pce.15667 (PMC12319266; doi:10.1111/pce.15667)
Supplement: Supplementary file 6 — Supmat. [file PCE-48-6941-s004.docx]

**SUPPORTING INFORMATION**

The following materials are available in the online version of this article.

**Supplemental Figure S1:** Multiple sequence alignment of the amino acid sequences of mature cFBP1 from pea (*Pisum sativum*) (NCBI accession number AAD10213.1), rapeseed (*Brassica napus*) (NCBI accession number NP_001302992.1), spinach (*Spinacia oleracea*) (NCBI accession number AAD10207.1) and Arabidopsis (*A. thaliana*) (NCBI accession number CAA41154.1), pig (*Sus scrofa*) fructose-1,6-bisphosphatase (FBPase) (NCBI accession number AAA31035.1) and cytosolic FBPase of Arabidopsis (cytFBPase) (NCBI accession number NP_175032.1) using Clustal Omega (<http://www.ebi.ac.uk/Tools/msa/clustalo/>). The numbers indicate the amino acid positions. Asterisks (*) indicate positions which have a highly conserved residue; Colon (:) indicates residues with strongly similar physiochemical properties; Period (.) indicates residues with weakly similar properties.

**Supplemental Figure S2: Stages in the construction of the promcFBP1:cFBP1_WT_, promcFBP1:cFBP1_C95S_, pDEST17- cFBP1_WT_ and pDEST17- cFBP1_C95S_ plasmids.** Plasmid constructs were produced using Gateway technology and confirmed by sequencing. Primers used for PCR amplification of complete a cFBP1 cDNA obtained from the RIKEN Arabidopsis cDNA collection (Seki et al., 1998, 2002) are listed in **Supplemental Table S1**.

**Supplemental Figure S3: C95S cFBP1 expression is sufficient to restore to WT the weak response of *cfbp1* plants to small microbial VCs**. (A) External phenotype and (B) rosette fresh weight (FW) of WT and *cfbp1* plants and WT and C95S cFBP1-expressing *cfbp1* plants grown in the absence or continuous presence of small fungal VCs for one week. Values in panel (B) are means▒±▒SE for three biological replicates (each a pool of 12 plants) obtained from four independent experiments. In (B), lowercase letters indicate significant differences, according to Student´s t-test (P▒<▒0.05) between: “a” VC non-treated WT plants and mutant plants, “b” VC-treated and non-treated plants, and “c” VC-treated WT and mutant plants.

**Supplemental Figure S4:** AlphaFold predicted conformational changes in the Mg^2+^- and FBP-binding domains. (A) Amino acids of the Mg^2+^ binding domains located within 5▒A from the Mg^2+^ ion in WT cFBP1 and C95S cFBP1. (B) Mg^2+^positions in the tetrameric in WT cFBP1 and C95S cFBP1. In (A), the two cFBP1 forms are presented as a ribbon diagrams (each cFBP1 form in a different color, for clarity) and Mg^2+^ ions are shown as pink (A) and green (B) spheres.

**REFERENCES TO SUPPLEMENTAL DATA**

**Seki M, Carninci P, Nishiyama Y, Hayashizaki Y, Shinozaki K** (1998) High-efficiency cloning of Arabidopsis full-length cDNA by biotinylated CAP trapper. Plant J **15**: 707–720

**Seki M, Narusaka M, Kamiya A, Ishida J, Satou M, Sakurai T, Nakajima M, Enju A, Akiyama K, Oono Y,** **et al** (2002) Functional annotation of a full-length Arabidopsis cDNA collection. Science **296**: 141–5
